# Rolling Locomotion of Cable-Driven Soft Spherical Tensegrity Robots

**DOI:** 10.1089/soro.2019.0056

**Published:** 2020-06-02

**Authors:** Kyunam Kim, Adrian K. Agogino, Alice M. Agogino

**Affiliations:** ^1^ Department of Aerospace, California Institute of Technology, Pasadena, California.; ^2^ NASA Ames Research Center, Moffett Field, California.; ^3^ Department of Mechanical Engineering, University of California at Berkeley, Berkeley, California.

**Keywords:** tensegrity robots, dynamic relaxation, greedy search, multigeneration Monte Carlo

## Abstract

Soft spherical tensegrity robots are novel steerable mobile robotic platforms that are compliant, lightweight, and robust. The geometry of these robots is suitable for rolling locomotion, and they achieve this motion by properly deforming their structures using carefully chosen actuation strategies. The objective of this work is to consolidate and add to our research to date on methods for realizing rolling locomotion of spherical tensegrity robots. To predict the deformation of tensegrity structures when their member forces are varied, we introduce a modified version of the dynamic relaxation technique and apply it to our tensegrity robots. In addition, we present two techniques to find desirable deformations and actuation strategies that would result in robust rolling locomotion of the robots. The first one relies on the greedy search that can quickly find solutions, and the second one uses a multigeneration Monte Carlo method that can find suboptimal solutions with a higher quality. The methods are illustrated and validated both in simulation and with our hardware robots, which show that our methods are viable means of realizing robust and steerable rolling locomotion of spherical tensegrity robots.

## Introduction

The term *tensegrity* was first coined by Fuller^1^ as a portmanteau of *tensional integrity*. The name comes from the unique design principle that tensegrity structures follow: structures are constructed with isolated rigid rods connected by a net of elastic cables providing tension to hold the structures. When an ideal tensegrity structure is loaded, both types of members bear loads only in axial directions; rods undergo pure compressive forces and cables pure tensile forces. By delicately balancing the cable tension forces and rod compression forces, the structure is able to maintain its shape without collapsing. The overall shape of the structure is determined by the distribution of internal forces across its members.

Tensegrity structures have several unique features that distinguish themselves from other structures.^2,3^ First, tensegrity structures are in general lightweight as they have minimal mass distribution in terms of load bearing since most of its internal volume is empty and the material is placed only on its load paths. This allows efficient use of material and results in a reduced weight of the structure for a given stiffness. Their structural level flexibility enables packing into small volumes for efficient transportation and on-site deployment by expanding back to their full volume with proper control of cable tensions. In addition, tensegrity structures can remain intact when overloaded or absorb impact shocks by exploiting their innate structural level compliance. The aforementioned properties of tensegrity structures are advantageous as a robotic platform, and the efforts have been made in the literature to transform different tensegrity structures into robots. These tensegrity robots constitute examples of soft robots that are characterized by elastic deformability attributable to the extensive use of deformable matter with little or no rigid material.^4^

Two of the earliest tensegrity robots introduced in the literature were based on tensegrity prisms with three or four rods, and it was shown that the robots can produce gaits using controllers developed with evolutionary algorithms.^5,6^ In addition, dynamic equations of motion of a similar three-rod tensegrity prism-based robot were developed and used to control the robot to follow simple geometric paths in a simulation environment.^7^ Since then, tensegrity robots were introduced in various forms. For instance, spine-like tensegrity robots were introduced with Central Pattern Generator-based controllers to realize crawling or snake-like motions,^8–11^ and legs were added to such robots later for legged locomotion.^12,13^ Yet other tensegrity robots based on multimodule tensegrity prisms were developed for duct exploration and maintenance tasks, and two prototypes were constructed for this purpose.^14,15^ Moreover, bioinspired tensegrity manipulators mimicking a human shoulder and elbow were developed^16,17^ by building upon the fact that tensegrity structures can effectively describe anatomical structures of the human body.^18,19^

**FIG. 1.**
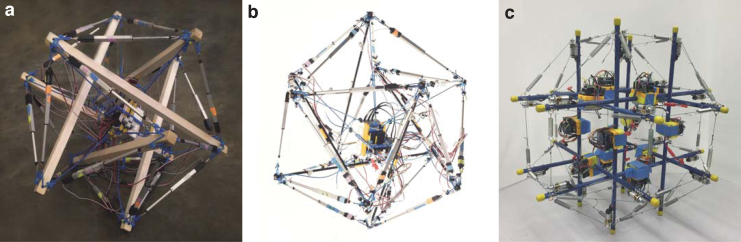
Rapidly prototyped tensegrity robots at the Berkeley Emergent Space Tensegrities lab, UC Berkeley. The first two robots **(a, b)** are based on a six-rod tensegrity structure, and the last one **(c)** is based on a twelve-rod tensegrity structure.^48^

Among the tensegrity robots that are being developed in various shapes, a special attention has been paid to *spherical* tensegrity robots as mobile robotic platforms.^20–24^ The term spherical tensegrity indicates tensegrity structures whose outer shapes are *similar* to a sphere and that are capable of rolling locomotion. While a spherical tensegrity structure with a small number of rods may seem to be a crude approximation of a sphere, its outer shape becomes a lot closer to a sphere when more rods are used to construct the structure (Fig. 1). A six-rod tensegrity structure that resembles an icosahedron is the simplest three-dimensional (3D) spherical tensegrity structure, and several robots have been introduced in the literature based on this structure.^20,21,23–30^

Some research on these robots developed locomotion strategies from trial-and-error hardware experiments,^25,26,31,32^ which can be time consuming and are not scalable to the robots with a larger number of members. To overcome this difficulty, other researchers developed locomotion strategies first in simulation, usually in conjunction with learning algorithms, to manage the inherent structural complexity of tensegrities, and then validated them with hardware robots.^28,33–38^ Most of the work taking this approach, however, relied on the outcome of the simulator and does not clearly reveal the underlying physics of how tensegrity robots locomote using structural deformation. Therefore, any unmodeled dynamics or interaction with environments that are not captured in simulation can possibly lead to a performance degradation of the developed strategies when they are implemented on the physical robots.

Recently, another mode of locomotion for a six-rod tensegrity robot driven by vibration was introduced, where high speed locomotion was achieved by properly tuning motor speeds to excite the robot structure at a maximum resonance.^39^ This approach, however, has a potential to fail on uneven or irregular terrains and has difficulties in precisely following desired paths or making turns, which would have been possible with rolling locomotion using structural deformation.

Relatively less work has been reported on sensing and state estimation of tensegrity robots. For example, a state estimation method using the Unscented Kalman Filter for fusing inertial measurement readings with radio time-of-flight range measurements was developed for a six-rod tensegrity robot.^40^ Another approach used liquid metal-embedded hyperelastic strain sensors^41^ to measure cable lengths of a tensegrity robot.^42,43^ Because the sensors were elastic, they also served as tension members of the robot and made extra cables unnecessary. This shows well the *redundancy* of tensegrity structures: members of tensegrity structures can simultaneously function as actuators or sensors in addition to being load-carrying members.^2,3^ Tensegrity structures were also proposed as sensors measuring forces and torques.^44,45^

### Our contribution

In this work, we present systematical ways of developing actuation strategies enabling rolling locomotion of spherical tensegrity robots and demonstrate them in simulation and with our hardware robots. By means of this work, we aim to consolidate and add to our research to date.^46–49^

We first revisit some of the concepts and techniques presented in our previous work. We start by breaking down the rolling motion of spherical tensegrity robots into a series of piecewise continuous motions, or *steps*, and classify them based on the geometry of the robots. The steps are realized by means of structural deformation, and therefore, we present in detail a tool to compute tensegrity deformations. At the core of our approach is the formulation of the dynamic relaxation, which is a numerical technique to efficiently solve for the deformation of tensegrity structures when actuation signals are given. We provide a set of equations that are used to numerically propagate system states and are singularity free. Hence, the formulation does not depend on a specific simulation environment and is applicable to other spherical tensegrity robots beyond the ones introduced in this work. The properties and implications of the dynamic relaxation are also discussed in detail, which were not presented in our previous work.

In addition, we reconsider two heuristic-based methods that find a set of desirable deformations leading to different types of steps: (1) a greedy search method that can quickly find solutions and (2) a multigeneration Monte Carlo (MGMC) based learning method that finds a set of suboptimal solutions for more reliable steps. By means of these methods, we obtain *actuation policies* that encode what actuation commands should be given to the robots to arrive at desirable deformations for making steps.

Our results are demonstrated and validated using the two different kinds of hardware spherical tensegrity robots we constructed: (1) six-rod tensegrity robots whose outer shapes are similar to the Jessen's orthogonal icosahedron^50^ and (2) a twelve-rod tensegrity robot whose outer shape is similar to a rhombicuboctahedron. To the best of authors' knowledge, the twelve-rod tensegrity robot was the first one of its kind introduced in the literature by the authors.^48,49^ In addition to the actuation policies for the six-rod tensegrity robots presented in our earlier work, the actuation policies for the twelve-rod tensegrity robot are newly presented in this work, which shows that our methods can work on different tensegrity systems. Furthermore, we newly provide a detailed comparison study of the greedy search and MGMC policies based on three criteria, namely, computation cost, robustness of steps, and required actuation energy.

### Notations

The notations used in this article are as follows. ℛ is the set of real numbers. ${ℛ}^{n}$ is the set of *n*-dimensional real vectors. Scalars are written in plain letters, vectors in small-bold letters, and matrices in capital-bold letters. *v_i_* is the *i*-th element of a vector v. 0 is a column vector of zeros with an appropriate dimension.

## Materials and Methods

### Geometries of spherical tensegrity robots

In this section, we introduce two different types of spherical tensegrity robots that are used as our test bed in this work. The first type of tensegrity robot consists of six rods and 24 cables and its geometry resembles the Jessen's orthogonal icosahedron (Fig. 1a, b). Each rod end, or *node*, of this tensegrity robot is connected to four neighbor nodes by cables, resulting in a structure with 8 equilateral and 12 isosceles triangles on the outer surface. Since there does not exist a cable connecting the edges between each pair of parallel rod ends, a total of six edges are missing cables. As a consequence, isosceles triangles also present themselves as *open triangles* that have cables only on two out of three edges. In contrast, every equilateral triangle is a *closed triangle* that has cables on all three edges. When the robot is standing on the ground, we assume only one triangle is in contact with the ground and call it as a *base triangle*.

A six-rod tensegrity structure is the simplest 3D tensegrity structure that is suitable for rolling locomotion. Although we show in the later sections that the robot based on this tensegrity structure can perform rolling, it does so in a zigzag way, not in a straight line, because its outer surface consists of triangles only. This behavior is acceptable for slow speed rolling, but it is not desirable for high speed rolling since the robot should change its moving direction after each and every *step* (i.e., a piecewise continuous motion between the exchange of two consecutive base triangles), which can cause loss of momentum from the robot.

To avoid the zigzag motion and to make straight rolling possible, another tensegrity robot whose outer surface mostly consists of rectangles is developed based on a twelve-rod tensegrity structure whose outer shape is similar to a rhombicuboctahedron. This is the simplest spherical tensegrity structure that has the desired outer surface property for straight rolling (Fig. 1c). Furthermore, the geometry is symmetric about three mutually-orthogonal planes when there is no deformation, and we exploit this feature in later sections when we obtain actuation strategies for the twelve-rod tensegrity robot. If the tensegrity deforms in a way that the symmetry about one of these planes is maintained, then the motion of the robot can be expressed on the two-dimensional plane by projecting its members onto that plane of symmetry, instead of describing the robot's motion in a 3D space as is the case of the six-rod robot. This allows reduction of the dimensions of state space and control inputs, which can be useful in designing controllers.

The outer surface of a twelve-rod robot consists of two types of rectangles and eight equilateral triangles. There are six *perpendicular rectangles* each of which is formed by nodes of four parallel rods and is perpendicular to the rods, and 12 *diagonal rectangles* each of which is formed by nodes of two orthogonal pairs of two parallel rods and is at an angle to the rods. As before, a *base rectangle* refers to a rectangle that is in contact with the ground. In general, we will use the term *base polygon* to indicate a polygon in ground contact.

### Hardware tensegrity robots

**FIG. 2.**
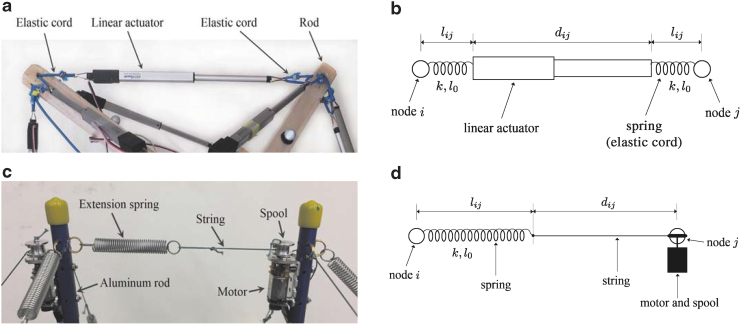
**(a, b)** Each edge of the six-rod robot consists of serial connections of two elastic cords and a linear actuator that controls the length and tension of the edge.^46^ **(c, d)** Each edge of the twelve-rod robot consists of an extension spring and a nonstretching string that is spooled in and out by a motor to control the edge length and tension.^48^

The hardware spherical tensegrity robots constructed based on the aforementioned geometries are shown in Figure 1. The first two robots consist of six rods and 24 edges, and the last robot is constructed with twelve rods with 48 edges. All rods and edges within each robot are identical. To minimize the weight of the robots, lightweight balsa wood or fiber glass rods are used for the six-rod robots, and hollow aluminum tubes are used for the twelve-rod robot. The total weights of the six- and twelve-rod robots are 2.7 and 1.8 kg, respectively. The rod lengths of the six- and twelve-rod robots are 65 and 45 cm, respectively, and all robots have the overall diameters of ∼1 m. All of our robots are cable driven meaning that the edge lengths are controlled for shape deformation while the rods do not have actuation and their lengths are constant. However, the edges of the robots are designed differently. Each edge of the six-rod robots is made up of two elastic cords and a linear actuator that are serially connected (Fig. 2a, b). In contrast, each edge of the twelve-rod robot consists of a nonstretching string that is connected to an extension spring. The other side of the string is connected to an actuated spool that controls the edge length and tension by winding or unwinding the string (Fig. 2c, d).

### Definition and classification of steps

**FIG. 3.**
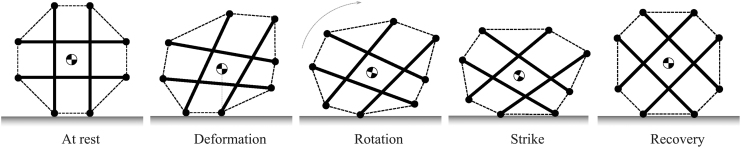
A two-dimensional conceptual diagram of different stages of a step. The robot deforms its structure using cable actuation and manipulates GCoM to leave the current base polygon to make a step. GCoM, ground projection of the center of mass.

Precision rolling of tensegrity robots is realized by a sequence of steps. A step of the spherical tensegrity robots is composed of five different stages as follows (Fig. 3):

1. At rest: The robot is not deformed and is at a neutral pose.
2. Deformation: The robot deforms by actuating some or all of its members in such a way that the ground projection of the center of mass (GCoM) escapes the current base polygon.
3. Rotation: The robot rotates about one edge of the base polygon.
4. Strike: The robot lands on the next base polygon.
5. Recovery: The robot recovers back to its neutral pose and prepares for the next step.

Strictly speaking, *rolling* is not a precise term to describe the motion of a spherical tensegrity robot because the motion is discontinuous with repeated impact events involved between the robot's rods and the ground. Moreover, the rods that are in ground contact are pivoted, and the contact points do not change during the piecewise continuous motion of the robot between impacts, whereas the ground contacting material point of a rolling rigid body continuously changes as the body rolls. For this reason, other terminologies were introduced in the literature to describe the motion, such as tipping over or tumbling. However, we continue to use the term rolling to describe the motion of spherical tensegrity robots for two reasons: (1) it is generally used and accepted in the field of tensegrity robotics, and (2) the motion becomes close to rolling as the number of rods constituting a spherical tensegrity structure grows. Sometimes the motion of spherical tensegrity robots is referred to as *punctuated rolling* to emphasize the discontinuous nature of the motion.

Using high symmetry of spherical tensegrity structures, their motion possibilities can be categorized into different types of steps based on the outer surface polygons involved during the steps. For instance, each closed triangle of the six-rod tensegrity robot is surrounded by three open triangles, and each open triangle is surrounded by two closed triangles and one open triangle. Because of this, the robot has to land on one of the three neighbor open triangles after a single step initiated from a closed base triangle. In contrast, the robot can land on either of the two neighbor closed triangles or on the neighbor open triangle after a single step initiated from an open base triangle. In summary, three different types of steps are possible:

- *CO-step*: Start from a *closed* base triangle and land on an adjacent *open* base triangle.
- *OC-step*: Start from an *open* base triangle and land on an adjacent *closed* base triangle.
- *OO-step*: Start from an *open* base triangle and land on an adjacent *open* base triangle.

A similar classification of steps is possible for the twelve-rod tensegrity robot. Recall that there are two types of base rectangles that the robot can stand on, namely, perpendicular and diagonal rectangles. We do not consider the case where the robot is standing on a triangle because this pose is not part of straight rolling. Then the following definitions of steps are possible from the robot:

- *PD-step*: Start from a *perpendicular* base rectangle and land on an adjacent *diagonal* base rectangle.
- *DP-step*: Start from a *diagonal* base rectangle and land on an adjacent *perpendicular* base rectangle.

### Form-finding of tensegrity robots

A compliant tensegrity robot makes a step by shifting its center of mass through structural deformation using actuation. To control the center of mass in a desired manner, a tool is necessary that can predict how tensegrity robots will deform as a response to the given actuation signals. Such a problem is called a *form-finding* problem of tensegrity structures.

Analytical modeling of tensegrity deformation is usually complicated due to complex interactions between highly interconnected members and is often limited to simple tensegrity structures with only a few members.^51–53^ To overcome this difficulty, the NASA Tensegrity Robotics Toolkit (NTRT)^54^ has been developed to provide the core methods to model, simulate, and control broad types of tensegrity robots. This open-source software package has been widely used in the field especially for the simulation of dynamic behavior of tensegrity robots, and its performance has been verified in the previous works of tensegrity robots.^11,13,14,28,37^

Since the form-finding problem is concerned about statics of tensegrities, we develop another tool based on the dynamic relaxation technique to solve the problem, which is grounded on Newton's law and numerically computes an equilibrium shape of a tensegrity robot for a given set of actuation commands. This approach admits explicit analytical expressions, and therefore, it does not rely on a specific simulation environment. In addition, the method is applicable to other tensegrity structures beyond the ones introduced in this work for computing their deformations.

In what follows, we formally establish the method in detail. The formulation assumes cable-actuated tensegrity robots whose edges change lengths for structural deformation while rigid rods have constant lengths.

### Dynamic relaxation with kinetic damping

Given a cable net structure whose initial configuration has nodes with unbalanced forces applied, the dynamic relaxation aims to find an equilibrium configuration at a minimum energy state in an iterative way.^55,56^ The technique uses either viscous or kinetic damping, and we choose to use the latter in this work considering that this type of damping is known to be convergent and stable for systems with large local disturbances^55^ as is the case with tensegrity robots.

The formulation of dynamic relaxation with kinetic damping starts from Newton's second law. First, each rod is modeled as two point masses located at the ends, and the masses are assumed to have a rigid and massless connection between them. Suppose a force $F_{i}\left(t\right)$ is applied to the *i*-th node whose mass is *m_i_*, where *t* denotes a time step. According to Newton's second law, the motion of the node is governed by:

(1) $$F_{i}\left(t\right)=m_{i}a_{i}\left(t\right),$$

where $a_{i}\left(t\right)$ is the acceleration of the node. The acceleration can be approximated using the centered finite difference form of the velocity as follows:

(2) $$a_{i}\left(t\right)={\dot{v}}_{i}\left(t\right)\approx \frac{v_{i}\left(t+\Delta t∕2\right)-v_{i}\left(t-\Delta t∕2\right)}{\Delta t},$$

where $v_{i}\left(t\right)$ is the velocity of the node and Δt is the time difference between the two consecutive updates. Combining Eqs. (1) and (2) results in an iterative form of the velocity update:

(3) $$v_{i}\left(t+\Delta t∕2\right)=v_{i}\left(t-\Delta t∕2\right)+\frac{\Delta t}{m_{i}}F_{i}\left(t\right).$$

Note that a fictitious value can be assigned to *m_i_*, which may or may not be taken from an actual physical system. Usually, *m_i_* and Δt are tuned for good convergence of the solution and numerical stability of the simulation.^55^ This way of choosing the figures may seem unreasonable at first sight; however, it is acceptable for the dynamic relaxation since the goal is to find the *final* shape of a tensegrity structure and the method is not concerned with the shape evolution between the initial and final shapes. As a result, the intermediate shape evolution obtained from the dynamic relaxation does not necessarily represent the actual dynamic behavior of the system.

From Eq. (3), the node velocity is updated for the following time step using the total node force $F_{i}\left(t\right)$ whose expression is provided in the following section. The node position $r_{i}\left(t\right)$ is updated using the updated velocity:

(4) $$r_{i}\left(t+\Delta t\right)=r_{i}\left(t\right)+v_{i}\left(t+\Delta t∕2\right)\Delta t.$$

The dynamic relaxation attempts to find an equilibrium of the structure from an arbitrary initial configuration whose initial node positions $r_{i}\left(0\right)$ for all *i* satisfy the rod length constraint. The initial node velocities are set to zero, that is, $v_{i}\left(0\right)=0,\forall i$. Because the centered finite difference form is used for the velocity, the first velocity update is slightly modified from Eq. (3):

(5) $$v_{i}\left(\Delta t∕2\right)=\frac{\Delta t}{2m_{i}}F_{i}\left(0\right).$$

In summary, the positions, velocities, and accelerations of all nodes can be computed for all future time steps starting from a given initial configuration if the node forces are tracked over time. This process is iterative because the node forces in turn are functions of node positions and velocities as will be shown later.

The kinetic energy of the system is another quantity of interest in using the dynamic relaxation with kinetic damping:

(6) $$KE\left(t\right)=\sum_{i=1}^{n_{n}}\frac{1}{2}m_{i}v_{i}\left(t\right)\cdot v_{i}\left(t\right),$$

where *n_n_* represents the total number of nodes. Unless the given initial configuration is already at an equilibrium state, some or all of the nodes experience unbalanced forces applied by the elastic tension members, and they start to move according to Eqs. (1)–(5), increasing the system's kinetic energy over time.

When we run the dynamic relaxation in our work, we assume that the actuation signals (i.e., lengths of rigid elements of the edges, namely, linear actuators of the six-rod robots and nonstretching strings of the twelve-rod robot) are given as some constants from either of the higher level greedy search or MGMC algorithms and assume there is no external forces applied to the system. Therefore, the only source of the incremental kinetic energy is the decrement of potential energy. The kinetic energy, however, does not monotonically increase over time because the system is a closed net structure and, after certain deformation, the nodes move in such a way that they decelerate and the kinetic energy starts to decrease as it transforms back to the potential energy. Hence, there exists a kinetic energy peak.

To bring the system to a minimum energy state, the kinetic energy is removed from the system at the peak by artificially setting all of the node velocities to zero. By doing so, the motion is instantaneously brought to a stop, and the system moves toward a minimum energy state, or an equilibrium, by dissipating the energy from the system. This interruption occurs at the peak to maximally remove the energy from the system and to minimize the number of such incidents. Then the above process restarts from the beginning with the new initial configuration defined as the last configuration at the energy peak. This iteration is repeated until the potential energy does not transform into kinetic energy anymore and the kinetic energy converges to zero, at which point we consider the system has reached its minimum energy state and we take the final shape as an equilibrium associated with the given actuation signals.

Notice that suddenly forcing the velocities to zero requires infinite accelerations and it cannot happen in real physical systems. Indeed, this is the reason why the intermediate deformation from the dynamic relaxation does not represent the real dynamic behavior of the system and only the final equilibrium configuration is physically meaningful. However, the removal of the kinetic energy allows one to quickly find a desired solution.

### Node forces

The node force $F_{i}\left(t\right)$ is a sum of two different types of forces: (1) $F_{i}^{s}\left(t\right)$, a sum of tensile forces applied by the elastic edges connected to the node, and (2) $F_{i}^{r}\left(t\right)$, a constraint force applied by the rigid rod connection to satisfy the constant rod length constraint. The total node force $F_{i}\left(t\right)$ is then:

(7) $$F_{i}\left(t\right)=F_{i}^{s}\left(t\right)+F_{i}^{r}\left(t\right).$$

Let 
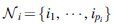
 be a set of neighbor nodes connected to the *i*-th node by edges, where *p_i_* is the total number of neighbor nodes of *i*. At each time step *t*, $F_{i}^{s}\left(t\right)$ is computed as the sum of individual edge forces:


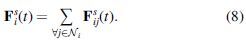


In Eq. (8), $F_{ij}^{s}\left(t\right)$ denotes the force exerted on node *i* at time *t* by the tension member located on the edge connecting nodes *i* and *j*. The description of this force depends on specific edge configurations. Two possible edge configurations are shown in Figure 2, which are used in our tensegrity robots. The first edge configuration has two identical springs (instantiated as elastic cords) at each end of the edge, and the two springs change the same in lengths when stretched by a linear actuator located at the middle of the edge. The second edge configuration connects a spring and a nonstretching string in series. The edge length is controlled by a motor placed at a node that winds or unwinds the string with a spool from the node.

In each of our robots, we used identical linear extension springs whose stiffness and rest length are denoted as *k* and *l*_0_, respectively. For the first edge configuration, we denote the stretched length of the spring as $l_{ij}$ and the controlled actuator length as $d_{ij}$. Then $F_{ij}^{s}\left(t\right)$ is computed as:

(9) $$F_{ij}^{s}\left(t\right)=\left\{\begin{array}{cc} k\left(l_{ij}\left(t\right)-l_{0}\right)\frac{r_{j}\left(t\right)-r_{i}\left(t\right)}{∥r_{j}\left(t\right)-r_{i}\left(t\right)∥} & ifl_{ij}\left(t\right)>l_{0}\\ 0 & ifl_{ij}\left(t\right)\leq l_{0} \end{array}\right.,$$

(10) $$l_{ij}\left(t\right)=\frac{1}{2}\left(∥r_{j}\left(t\right)-r_{i}\left(t\right)∥-d_{ij}\right).$$

Note that the edge tension is set to zero in Eq. (9) when the extension spring becomes shorter than its rest length. Physically, this implies that the edge is slack.

For the second edge configuration, we denote the stretched spring length and the controlled string length as $l_{ij}\left(t\right)$ and $d_{ij}$, respectively. The tension on this edge is computed from Eq. (9) with $l_{ij}\left(t\right)$ defined as

(11) $$l_{ij}\left(t\right)=∥r_{j}\left(t\right)-r_{i}\left(t\right)∥-d_{ij}.$$

Notice that the actuation signal $d_{ij}$ is independent of time and is a constant in Eqs. (10) and (11). The value of $d_{ij}$ is either selected by the greedy search algorithm or randomly sampled from MGMC as described in the later sections, and it is treated as a constant while running the dynamic relaxation to seek an equilibrium configuration for a given set of actuation signals. In other words, the set of actuation signals $d_{ij}$ for all edges is considered as the source of structural deformation, and it serves as a constant input to the dynamic relaxation.

The rod constraint force $F_{i}^{r}\left(t\right)$ uniquely appears in tensegrity structures unlike $F_{i}^{s}\left(t\right)$, which also appears in pure tensile structures. The correct description of this force is necessary to ensure the constant distance between the two end nodes of a rigid rod and to guarantee the integrity of the whole structure.

We use the following coordinate systems to develop the constraint force expression. The position of the first rod end node is defined as $r_{i}\left(t\right)$ using the Cartesian coordinate system in the inertial frame. The position of the other node is defined relative to the first one as $r_{k}\left(t\right)=r_{i}\left(t\right)+R_{0}e_{R_{ik}}\left(t\right)$ using a spherical coordinate system with a set of right-handed orthonormal basis vectors 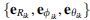, where *R*_0_ represents the constrained rod length. The constraint forces acting on the rod end nodes *i* and *k* are:

(12) $$F_{i}^{r}\left(t\right)=-F_{ik}^{r}\left(t\right)e_{R_{ik}}\left(t\right),F_{k}^{r}\left(t\right)=F_{ik}^{r}\left(t\right)e_{R_{ik}}\left(t\right),$$

(13) $$F_{ik}^{r}\left(t\right)=\left(ma_{i}\left(t\right)-F_{k}^{s}\left(t\right)\right)\cdot e_{R_{ik}}\left(t\right)-mR_{0}^{-1}∥v_{k}\left(t\right)-v_{i}\left(t\right){∥}_{2}^{2},$$

(14) $$e_{R_{ik}}\left(t\right)=\frac{r_{k}\left(t\right)-r_{i}\left(t\right)}{∥r_{k}\left(t\right)-r_{i}\left(t\right)∥}.$$

In Eq. (13), we assume that the node masses are equal (i.e., $m_{i}=m_{k}=m$) due to the symmetry. This equation is obtained from Lagrange's equations of motion with the consideration of the rod constraint. Note that Eq. (13) is easily computed using r, v, and a that are obtained from Eq. (2) to (4). It is also singularity free since $r_{i}\neq r_{k}$ for i≠k and is numerically stable. As a result, the rod constraint forces $F_{i}^{r}\left(t\right)$ and $F_{k}^{r}\left(t\right)$ can be updated over time using Eqs. (12)–(14).

### Actuation policies

If different actuator signals are provided as inputs to the dynamic relaxation, the resulting equilibrium configurations would also be different. Clearly, not all equilibrium configurations would allow the robots to make a step, and the actuator signals have to be chosen carefully to realize a successful step. Hence, we want to find *actuation policies* that will deform the tensegrity robots in a favorable way for realizing steps. Precisely, we define an actuation policy as a set of actuation commands that should be given to the actuators controlling the edge lengths to realize steps. We present two methods for obtaining valid actuation policies: a greedy search algorithm and a MGMC based learning algorithm. Both algorithms use the heuristic derived from a physical consideration, which makes them a valid approach for the development of actuation policies for general spherical tensegrity robots.

### Greedy search method

The greedy search can find solutions, even for problems of high complexity, in a reasonable amount of time when good heuristics are used.^57^ The method can quickly find actuation policies, and thus, it is suitable for checking the feasibility of steps or obtaining actuation policies that can serve as an initial solution to a more sophisticated optimization-based approach. Furthermore, this method is less computationally intense than MGMC, and it can be implemented on a resource-constrained on-board computer to (re-)compute actuation policies of the robots when the previous actuation policies become not valid anymore; such a case may arise, for instance, when the hardware experienced major changes such as cable breakage after deployment. The solutions found with this method are not optimal, but they turn out to be successful actuation policies for our robots.

**FIG. 4.**
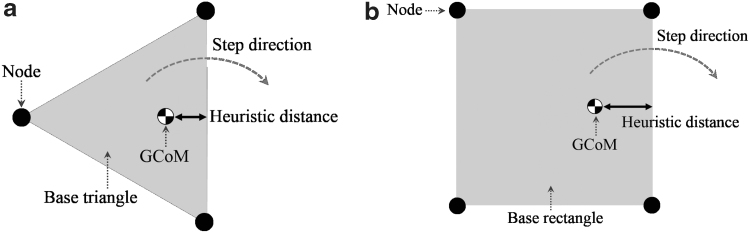
Definition of the heuristic distances used for the greedy search and MGMC. **(a)** Six-rod robot, **(b)** twelve-rod robot. MGMC, Multigeneration Monte Carlo.

The greedy search algorithm is implemented as follows. First, we define our heuristic function as the distance between the robot's GCoM and one of the base polygon's edges serving as the rotation axis about which a step will be made (Fig. 4). The heuristic is defined in this way from the physical observation that the robot will become unstable and perform a step when its GCoM is placed outside of the base polygon. We let the heuristic be positive (or negative) if GCoM is inside (or outside) of the base polygon after deformation. When GCoM is right on the edge, the heuristic becomes zero and the robot initiates the rotation stage of a step, from which point further deformation would lead to a successful completion of the step.

The search process begins with the initial robot state being neutral without any deformation. The base polygon and rotation axis are automatically selected by the algorithm once the final deformation is computed from the dynamic relaxation such that the resulting heuristic is minimum among all possible projections of the center of mass to outer surface polygons. Because our tensegrity robots, regardless of the choice of the base polygons and rotation axes, place their GCoM within their base polygons when not deformed, the search starts from a positive heuristic value. The leaf nodes^4^ of the search tree are expanded by changing the edge lengths one at a time by a prespecified amount. For the sake of simplicity and speeding up the search process, we only consider binary states, namely, maximum and minimum edge lengths. Although the assumption is seemingly restrictive, we were able to find successful actuation policies for all types of steps for our six- and twelve-rod tensegrity robots. Note that higher resolution actuation policies are always obtainable by allowing finer edge length changes at the cost of increased computation.

From the initially neutral robot, the first expansion of the search tree is done by fully retracting each one of *n_a_* edges and generating *n_a_* leaf nodes, where *n_a_* represents the total number of actuated edges. The robot's deformations under these actuation signals are computed using the dynamic relaxation, and each obtained deformation is evaluated using the heuristic. Then the leaf node with the smallest heuristic value after the first expansion is identified, and the second expansion is done from the node by fully retracting each one of the remaining $n_{a}-1$ edges in addition to the retraction of the first edge that the node already included. The newly generated leaf nodes are then evaluated, and the next round of expansion happens from the node that has the least heuristic value among all the nodes generated so far. Again, the expansion is done by fully retracting each one of the remaining edges in addition to the edges that the current node already included. This procedure is repeated until the leaf node with its heuristic value less than or equal to zero is found, and the goal node is taken as our actuation policy for the step. The search is run independently for different types of steps to develop their respective actuation policies.

### MGMC-based learning method

Although the greedy search method is effective and efficient in developing actuation policies, it has a couple of limitations. The first is that the method returns only one solution per run, and therefore, it is not possible to examine the quality of the solution or reliability of the step occurring from this solution. Another downside is that the search space becomes extremely large when we attempt to increase the actuation resolution from binary to a larger number. To overcome these barriers, we propose an evolutionary approach based on Monte Carlo sampling, with the goal of finding suboptimal equilibrium configurations that result in the most negative heuristic values possible. We prefer to have large negative heuristic values as they imply more instability of the deformed robot, which in turn imply more reliable steps. The solutions are suboptimal in the sense that the method is not guaranteed to find an equilibrium configuration with the absolute minimum heuristic value, but its solutions are optimal within the sample set. Indeed, it turns out that these solutions are of higher quality than the deformations found in the greedy approach. In our formulation, the sample corresponds to actuation signals, and we use the dynamic relaxation technique to solve for the resultant equilibrium shape. The evaluation of each sample is done using the same heuristic as before (Fig. 4). Furthermore, sampling in the current generation is done in the neighborhood of the best sample from the previous generation, with the expectation of getting higher quality samples as the generation evolves.

### Sampling of equilibrium configurations

Let 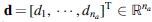 be a vector of the lengths of edge linear actuators or actuated edge strings as in Figure 2, where *d_i_* is the target length of the *i*-th actuator or string and *n_a_* is the total number of actuated edges in the robot. To find desirable equilibrium configurations, a number of instances of the vector d are sampled by sampling each component *d_i_* independently from a uniform distribution within a physically acceptable range. For each sampled d, the dynamic relaxation is run from a neutral initial configuration, and the resulting equilibrium configuration is found and evaluated using the heuristic. To maximize the utility of the samples and minimize the number of samples collected, we do not specify which outer surface polygon acts as the base polygon and which one of its edges serves as a rotation axis of the step for the found equilibrium configuration. Instead, the center of mass is projected as a candidate GCoM onto outer surface polygons that are the same type as the base polygon of the step for which the policy is sought, and for all projections, the distances between the projected points and the edges of the projection polygons are checked. The minimum of these values is assigned as the final heuristic value of that configuration. This process is repeated over a large number of samples, and the one with the minimum heuristic among the set of samples is identified as the best configuration of the set.

Because of the high dimension *n_a_* of the sampled vectors d and the wide interval of the sampling space, it is unlikely that a single run of Monte Carlo will find a desired solution. In the multigeneration scheme, the best performing sample from the previous generation is saved, and new samples are generated from a close neighborhood of this sample. As the generation progresses, we expect that the majority of our new samples will be sampled from high performing regions of the sampling space.

For the samples of the first generation, elements of the vector $d_{1}$, whose subscript denotes its generation, are all independently sampled from a uniform distribution of $\left[d_{\min},d_{\max}\right]$, where $d_{\min}$ and $d_{\max}$ represent minimum and maximum lengths, respectively. After obtaining all of the first generation samples and evaluating them with the heuristic, the best configuration 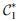 with the minimum heuristic value and the sampled actuation signal vector $d_{1}^{*}$ that produced this configuration are identified. For the subsequent generations $j=2,3,\cdots ,d_{j}$ is sampled around the best sample from the previous generation, $d_{j-1}^{*}$. Specifically, a number of samples $d_{j}$ are drawn from a uniform distribution of $\left[d_{j-1}^{*}-\delta d,d_{j-1}^{*}+\delta d\right]$, where 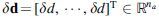 is a constant vector defining a neighbor region. Once a desired number of samples are obtained and evaluated at the *j*-th generation, the configuration with the minimum heuristic $C_{j}^{*}$ and the sample vector $d_{j}^{*}$ producing this configuration are identified. In the (j+1)-th generation, $d_{j+1}$ is sampled around $d_{j}^{*}$ in a similar manner, and the process is repeated until termination conditions are met or the predefined maximum number of generations or samples is reached.

## Results

In this section, we use the methods presented in the Materials and Methods section to compute actuation policies for our tensegrity robots. First, we apply the greedy search method to the six-rod and twelve-rod tensegrity robots to obtain their actuation policies for all possible types of steps. We also develop the actuation policies for the same robots using MGMC and compare them against the ones computed by the greedy search. To prove the efficacy of our methods, the actuation policies for the six-rod tensegrity robot are implemented and validated on the hardware robots, and the results are presented.

### Actuation policy for six-rod tensegrity robot using greedy search

When the six-rod tensegrity robot is standing on one of its closed triangles, it can make a CO-step in three different directions and each step uses one of the base triangle's edges as a rotation axis. Because of the threefold symmetry of the six-rod tensegrity robot, actuation policies for the three CO-steps are also symmetric, and therefore, it suffices to find an actuation policy for only one of the three CO-steps. Indeed, the actuation policies for all other CO-steps of the robot can easily be inferred from this result by exploiting the structural symmetry.

The six-rod tensegrity robot is balanced better when its base triangle is a closed triangle than the case of an open triangle. This happens because open triangles have narrower widths and smaller areas than those of closed triangles due to the missing edges, and the heuristic value is smaller for open base triangles than closed base triangles. For this reason, the six-rod tensegrity robot lands on a closed base triangle in most cases during rolling locomotion, but there are situations when the robot lands on an open base triangle due to instabilities or interaction with the ground. Two different types of steps are available from this pose: OC- and OO-steps. As in the case of CO-steps, it suffices to obtain one representative actuation policy per OC- and OO-steps, and the actuation policies for all other steps of these kinds can be obtained from the structural symmetry. Therefore, we seek one actuation policy for each type of steps.

**FIG. 5.**
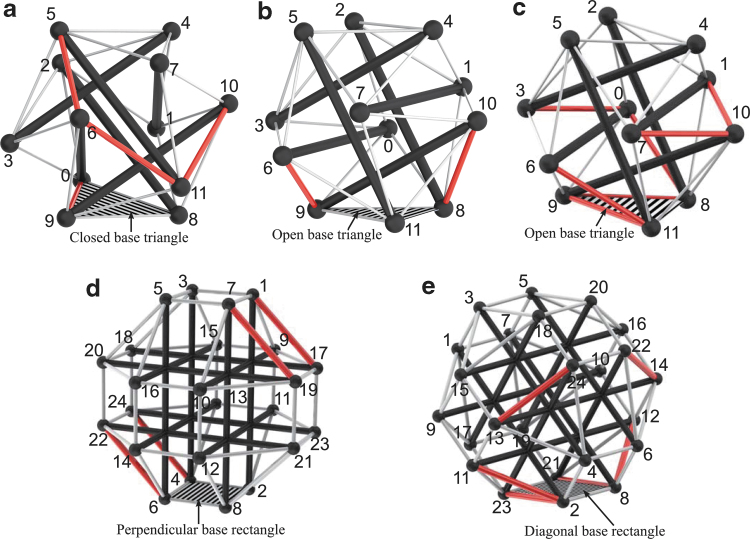
Actuation policies found with the greedy search algorithm, **(a)** CO-step, **(b)** OC-step, **(c)** OO-step, **(d)** PD-step, and **(e)** DP- step. Hashed polygons are base polygons, and thick *red* edges are actuated cables. Node numbers used in Table 1 are also shown.

The greedy search algorithm was implemented in MATLAB using the parameters measured from our hardware robots. Initially, the lengths of all linear actuators were set to 30 cm, and the ones chosen by the algorithm when expanding the leaf nodes were retracted to 20 cm. The rod length was 65 cm, spring rest length was 3.8 cm, and spring stiffness was 1193 N/m. The obtained actuation policies for CO-, OC-, and OO-steps are reported in Table 1 and Figure 5. When the CO-step actuation policy was forward simulated in NTRT, a CO-step was automatically followed by an OC-step, and the simulated robot arrived at the next closed base triangle. In other words, the actuation policy resulted in a closed to open to closed base triangle step or a *COC-step* and skipped the recovery stage of the CO-step. The COC-step was possible because the deformation by the CO-step policy allowed GCoM to cross the narrow width of the intermediate open triangle and enabled an OC-step without further deformation. In addition, the momentum of the robot gained from the CO-step was also providing some assistance.

**Table 1.** Actuation Policies Found with the Greedy Search

Type of step	Starting polygon	Landing polygon	Actuation policy
CO-step	(0,8,9)	(6,9,11)	(0,9), (5,6), (6,11), (10,11)
OC-step	(8,9,11)	(0,8,9)	(6,9), (8,10)
OO-step	(8,9,11)	(8,10,11)	(8,9), (9,11), (7,10), (1,10), (6,11), (0,8), (0,3)
PD-step	(2,4,6,8)	(2,8,21,23)	(4,24), (6,22), (1,17), (7,19)
DP-step	(2,8,21,23)	(2,4,6,8)	(2,23), (8,21), (2,11), (8,12), (13,24), (14,22)

Numbers represent node numbers, see Figure 5.

Two-tuples represent actuated edges defined by their two end nodes.

Three-tuples represent triangles formed by the three vertex nodes.

Four-tuples represent rectangles formed by the four vertex nodes.

For the definitions of types of the steps, see Materials and Methods section.

Note that the number of actuators included in the OC-step policy is less compared with the CO-step policy, which implies that less actuation effort is required to make an OC-step compared to a CO-step. This agrees with our previous observation: the open triangles provide less static balance compared to the closed triangles, and less deformation is sufficient to make an OC-step. However, the OO-step policy includes seven actuators and is more challenging and energy inefficient compared to CO- and OC-steps. Therefore, it is better to avoid this step whenever possible.

**FIG. 6.**
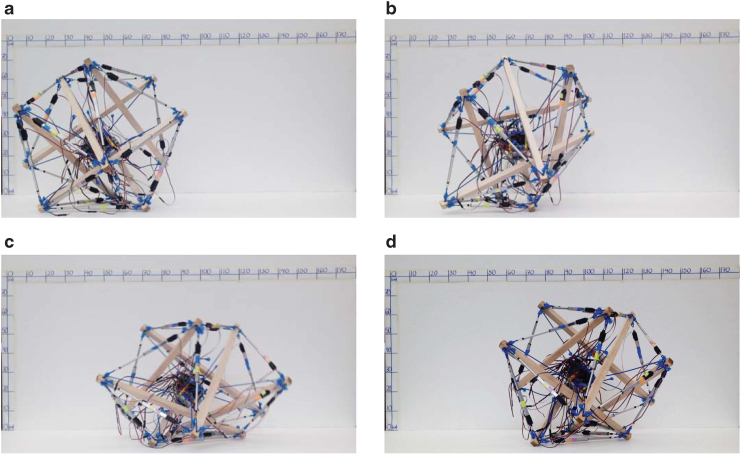
Still images of the six-rod tensegrity robot performing one COC-step. The robot starts with a CO-step, and the following OC-step is automatically performed.^46^ **(a)** Initially at rest, **(b)** deformation, **(c)** rotation and strike, **(d)** recovery.

To physically demonstrate and validate the obtained actuation policies, we implemented and tested them on our six-rod tensegrity robot. We attempted to realize all possible CO-steps (three from each of the eight closed triangles) and OC-steps (two from each of the twelve open triangles) using the respective actuation policies, and each step was performed thrice for a consistency check. We observed from our experiments that all of the steps were successful and all of the CO-steps resulted in COC-steps as predicted in the simulation. Figure 6 shows the robot performing one COC-step.

**FIG. 7.**
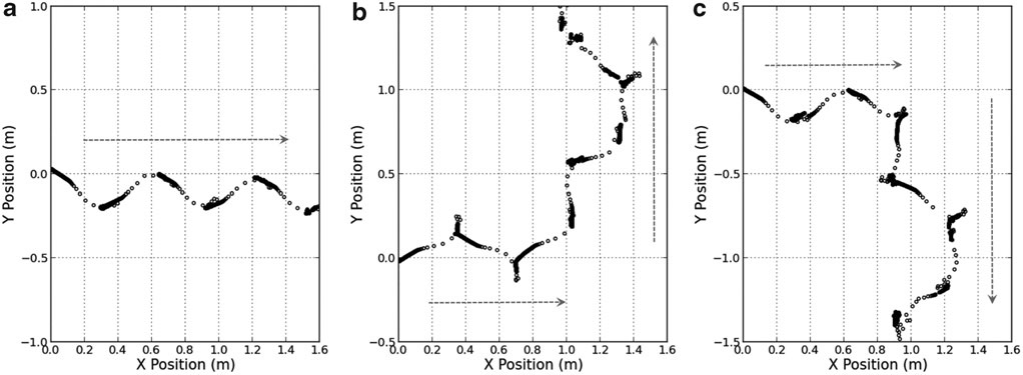
GCoM trajectories tracked with a motion capture system during the robot locomotion.^46^ Markers represent positions of GCoM for every 0.1 s, **(a)** move forward, **(b)** turn left, **(c)** turn right.

More generalized motions of the robot, such as moving forward or turning left or right, can be performed by linking together appropriate COC-steps as shown in Figure 7, where the GCoM trajectories tracked by a motion capture system are shown. In Figure 7a, the robot starts from the origin and moves forward in a zigzag manner by making five COC-steps. In Figure 7b and c, the robot starts from the origin, moves forward by taking three COC-steps, and then changes its heading direction at the fourth step, which shows that the robot is steerable. These basic motions could serve as motion primitives of the robot and may be combined together to create more complex paths. Finally, we note that the motions required only CO- and OC-steps and no OO-steps were needed; hence, omitting OO-steps does not restrict general motion of the robot.

### Actuation policy for six-rod tensegrity robot using MGMC

In this section, we seek an actuation policy for a CO-step of the six-rod tensegrity robot using the MGMC method implemented in MATLAB. For this, 30 generations were run with 500 samples per generation, and the simulation used parameters from the physical robot. Specifically, $n_{a}=24$ because all of the robot edges have actuation, and the elements of the vector $d_{j}\in {ℛ}^{24}$ were uniformly sampled from $\left[d_{\min},d_{\max}\right]$ with $d_{\min}=20$ cm, $d_{\max}=30$ cm, and δd=1 cm.

**FIG. 8.**
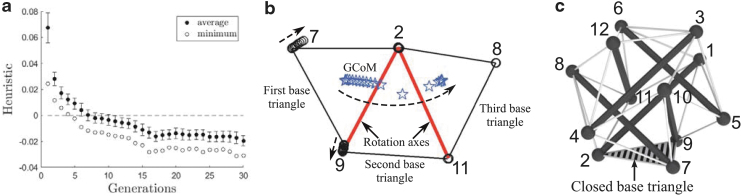
**(a)** Evolution of average and minimum heuristic values over 30 generations. Error bars represent standard deviations. The existence of the samples with negative heuristic values proves that MGMC found higher quality actuation policies than the greedy search whose best deformation had minimum heuristic equal to zero. **(b)** Trajectories of base *triangle* nodes and GCoM when the six-rod robot is deformed with the actuation policy provided in Table 2. *Black circles* are node trajectories, and *blue stars* are GCoM trajectories. *Thick red lines* are rotation axes. *Triangles* are base *triangles* that the robot crossed over while performing the step. **(c)** Node numbers.

**FIG. 9.**
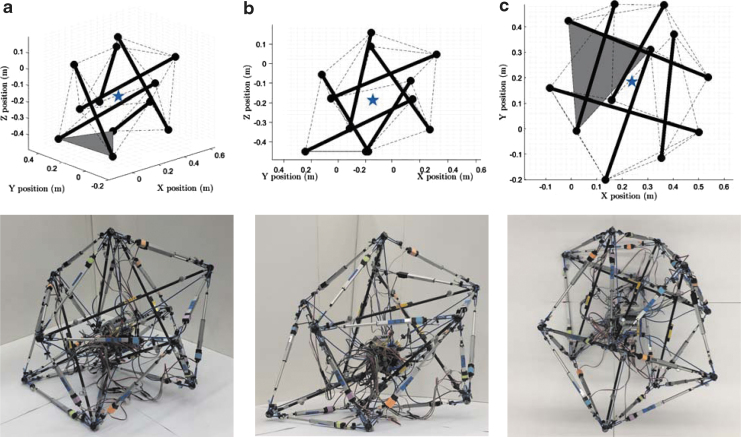
The best shape with the minimum heuristic value out of all samples obtained by MGMC, **(a)** perspective, **(b)** side, and **(c)** top. The figures on the *top row* are simulation results, and the figures on the *bottom row* show deformation of our physical robot. In the simulation figures, *thick black lines* are rods, thin *dashed lines* are edges, *blue stars* are centers of mass, and *gray triangles* are base *triangles*, respectively.^47^

The evolution of minimum and average heuristic values over generations is plotted in Figure 8a. For the first few generations, all of the sampled configurations had positive heuristic values, but the number of samples with negative heuristic increased as the generations progressed. Moreover, both the minimum and average values decreased as the generations evolved, and they converged after generation 20. The best configuration of all the samples was found in generation 29, and its heuristic value was −0.031 meaning that GCoM was placed 3.1 cm away from the base triangle. This configuration is shown in different perspectives in Figure 9. The sampled actuation signal vector that resulted in this configuration is taken as our actuation policy and is provided in Table 2.

**Table 2.** Actuation Policy for a CO-Step Obtained with Multigeneration Monte Carlo

Actuated edge	(1,5)	(1,6)	(1,9)	(1,11)	(2,7)	(2,8)	(2,9)	(2,11)
Actuator length (cm)	29.1	30.0	29.6	20.7	20.0	20.1	29.4	29.8
Actuated edge	(3,5)	(3,6)	(3,10)	(3,12)	(4,7)	(4,8)	(4,10)	(4,12)
Actuator length (cm)	26.1	25.1	29.7	21.4	25.1	20.4	20.4	29.1
Actuated edge	(5,9)	(5,10)	(6,11)	(6,12)	(7,9)	(7,10)	(8,11)	(8,12)
Actuator length (cm)	20.5	29.3	28.5	25.9	29.4	29.9	24.3	20.3

Two-tuples represent actuated edges defined by their two end nodes.

Node numbers follow Figure 8c.

The obtained actuation policy was tested on the hardware robot. The comparison of the deformation between the simulation and hardware is presented in Table 3, which shows that the actual deformation of our physical robot closely followed what was predicted in the simulation with a maximum of 5.25% error in final edge lengths. The robot was able to perform a COC-step with this actuation policy, and the GCoM trajectory of a single COC-step tracked with a motion capture system is shown in Figure 8b.

**Table 3.** Comparison of Edge Lengths Between Simulation and Hardware Experiment

Edge	Simulated lengths (cm)	Measured lengths (cm)	Error (%)
(1,5)	43.1	43.2	0.232
(1,6)	43.3	44.6	3.002
(1,9)	42.5	42.1	0.941
(1,11)	34.2	34.5	0.877
(2,7)	34.0	33.5	1.471
(2,8)	36.6	35.3	3.552
(2,9)	43.2	44.1	2.083
(2,11)	44.3	44.9	1.354
(3,5)	41.7	40.4	3.118
(3,6)	38.1	40.1	5.249
(3,10)	43.4	43.3	0.230
(3,12)	36.3	36.3	0
(4,7)	39.8	40.1	0.754
(4,8)	36.4	35.4	2.747
(4,10)	33.5	34.8	3.881
(4,12)	43.6	43.4	0.459
(5,9)	33.6	34.2	1.786
(5,10)	43.4	44.3	2.074
(6,11)	42.3	41.8	1.182
(6,12)	39.9	40.3	1.003
(7,9)	43.3	43.9	1.386
(7,10)	43.7	43.7	0
(8,11)	39.7	39.2	1.259
(8,12)	34.6	35.2	1.734

Two-tuples represent edges defined by their two end nodes.

Node numbers follow Figure 8c.

### Actuation policy for twelve-rod tensegrity robot using greedy search

The twelve-rod tensegrity robot has an outer shape similar to a rhombicuboctahedron that is symmetric about three mutually-orthogonal planes (Fig. 1c). Two of these planes are also perpendicular to the ground, and the robot can make steps in a direction that is contained in either of the planes. We choose one of the two planes and define it as a sagittal plane of the robot in which the desired step direction is contained. Then we develop actuation policies that are symmetric about this sagittal plane, that is, a pair of cables that are mirrored about this plane are actuated in the same way. Note that the search space (or the sampling space in the case of MGMC) is greatly reduced by taking this approach. This is the advantage of the symmetry of the twelve-rod tensegrity robot that we discussed earlier in the Geometries of Spherical Tensegrity Robots section.

When the twelve-rod tensegrity robot is standing on one of its perpendicular rectangles, it can make a PD-step in four different directions. In contrast, it can make a DP-step in two opposite directions when starting from one of its diagonal rectangles. Similar to the six-rod tensegrity robot case, a single PD- or DP-step actuation policy can be extended to all other PD- and DP-step policies with the help of the structural symmetry of the robot, and therefore, we seek one representative actuation policy per step.

The greedy search algorithm was run in MATLAB. The rod length was 45 cm, and the edge string lengths were set to 16 cm initially and pulled to 8 cm if they were chosen by the algorithm for expanding the leaf nodes. The spring rest length was 3.8 cm, and the spring stiffness was 770.6 N/m. The obtained actuation policies for PD- and DP-steps are reported in Table 1 and Figure 5. Note that the number of actuators included in the DP-step policy is greater compared with the PD-step policy. This implies that the diagonal base rectangles provide better static balance than the perpendicular rectangles, and hence, DP-steps require more actuation effort.

### Actuation policy for twelve-rod tensegrity robot using MGMC

Next, we compute PD- and DP-step actuation policies using the MGMC method. Since there exist a total of 20 edges on each side of the sagittal plane, the dimension of each sampled vector $d_{j}$ is also $n_{a}=20$, assuming that all of them are independently actuated.

The MGMC algorithm was run twice to separately obtain PD- and DP-step actuation policies using the physical parameters measured from our hardware robot. Initially, the structure was set to neutral with no deformation, and all of the edge string lengths were set to 12 cm. The motors installed on the robot can spool in (resp. out) the strings to the minimum (resp. maximum) length of $d_{\min}=8$ cm (resp. $d_{\max}=16$ cm) without significantly loading themselves, and these numbers are used in the simulation. For the first generation samples, the elements of $d_{1}\in {ℛ}^{20}$ were sampled from a uniform distribution of $\left[d_{\min},d_{\max}\right]$. Then δd was set to 1 cm for all later generations such that the sampling is done within a close neighborhood of the best sample from the previous generation. A total of 25 generations were run, and 100 samples were obtained per generation.

**FIG. 10.**
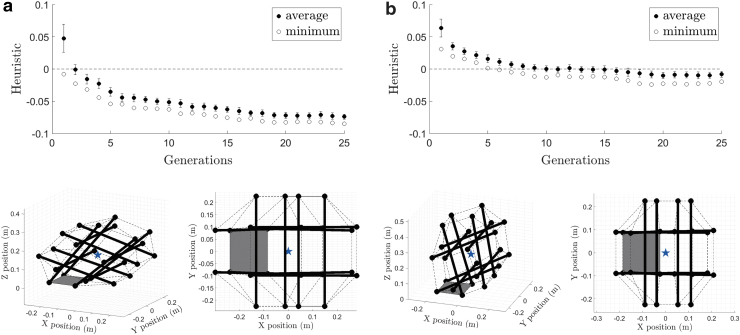
**(a)** PD-step policy simulation. **(b)** DP-step policy simulation. The *top row* figures are evolution of average and minimum heuristic values over 25 generations with 100 samples per generation. Error bars represent standard deviations. The best equilibrium configurations of the twelve-rod robot are shown in the *bottom row* from perspective (*left*) and top (*right*) views. In both cases, GCoM (*blue star*) is located outside of the *gray* base rectangle, and the robot is able to make steps with these deformations.

The evolution of average and minimum heuristic values over generations for PD- and DP-step simulations is presented in Figure 10. In both cases, the heuristic values converged toward the end of the simulation, and successful actuation policies were found after a sufficient number of generations. A comparison of the minimum heuristic values of the PD- and DP-step policies reveals that PD-steps are more reliable and easier to perform than DP-steps. From each simulation, the best sample with the minimum heuristic among all the samples is taken as the actuation policy shown in Table 4, and the resulting deformations are depicted in Figure 10.

**Table 4.** Actuation Policies for PD- and DP-Steps Obtained with Multigeneration Monte Carlo


Actuated edge	(1,3), (5,7)	(3,18), (5,20)	(18,24), (20,22)	(4,24), (6,22)	(2,4), (6,8)
PD string length (cm)	16.0	15.1	13.8	8.1	8.3
DP string length (cm)	15.6	10.8	15.6	8.7	8.3
Actuated edge	(2,23), (8,21)	(17,23), (19,21)	(1,17), (7,19)	(1,9), (7,10)	(15,18), (16,20)
PD string length (cm)	16.0	13.6	8.0	15.3	9.1
DP string length (cm)	8.4	16.0	15.9	15.7	14.0
Actuated edge	(4,13), (6,14)	(11,23), (12,21)	(3,15), (5,16)	(2,11), (8,12)	(13,24), (14,22)
PD string length (cm)	15.2	8.7	8.2	12.0	16.0
DP string length (cm)	10.0	15.3	12.8	8.5	8.1
Actuated edge	(9,17), (10,19)	(9,11), (10,12)	(11,13), (12,14)	(13,15), (14,16)	(9,15), (10,16)
PD string length (cm)	8.3	9.4	12.5	13.5	10.1
DP string length (cm)	12.8	15.3	12.2	10.8	9.9

Two-tuples represent actuated edges defined by their two end nodes.

Node numbers follow Figure 5d for PD-policy and Figure 5e for DP-policy.

### Comparison of actuation policies by greedy search and MGMC

We compare the actuation policies obtained by the two methods from the following three aspects: (1) computation cost, (2) robustness of steps, and (3) required actuation energy.

In our greedy search simulations, the total numbers of evaluated configurations were as follows: 72 for CO-policy, 34 for OC-policy, 145 for OO-policy, 24 for PD-policy, and 45 for DP-policy. These numbers are significantly smaller than the total numbers of evaluated samples by the MGMC method: 15,000 for CO-policy and 2500 for PD- and DP-policies. Hence, the greedy search method was able to find feasible solutions much faster than MGMC as discussed earlier.

The heuristic values of the actuation policies found by the greedy search were: 0 for CO-policy, −0.004 for PD-policy, and −0.015 for DP-policy. In contrast, the heuristic values of the actuation policies from MGMC were: −0.031 for CO-policy, −0.085 for PD-policy, and −0.024 for DP-policy. In all cases, the quality of the MGMC policies is better than the greedy search counterparts, and the steps made with the former would be more reliable than those made with the latter.

Finally, we compare how much actuation energy is required to perform each type of steps with the obtained actuation policies. For this, let us define the total potential energy of the robot at equilibrium as the sum of all potential energies of elastic members:


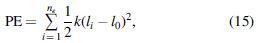


where *n_e_* is the total number of elastic members, and *k*, *l_i_*, and *l*_0_ are the stiffness, stretched length, and rest length of the *i*-th elastic member. In all of our simulations, no elastic member was slack and $l_{i}>l_{0},\forall i$. When the robots are deformed according to the actuation policies, actuators supply energy to the robots for the deformations to happen. Based on this observation, the actuation energy required by the actuation policies is computed as the potential energy difference before and after the deformation. From our simulation results, the energies required by the greedy search policies were 26.44 J for CO-policy, 19.22 J for PD-policy, and 36.50 J for DP-policy and by the MGMC policies were 17.07 J for CO-policy, 11.53 J for PD-policy, and 20.23 J for DP-policy. Note that the MGMC policies require less actuation energy than the greedy search counterparts, and hence, the former is more energy efficient. In addition, the DP-policies from both methods require more energy than the PD-policies, which confirms our earlier result that DP-steps are harder to make than PD-steps.

In conclusion, when there exists a sufficient amount of time and computational resources to compute actuation policies, it is better to run the MGMC method as it provides actuation policies that would result in more reliable steps with less actuation energy. However, the greedy search method is better when there is a need to rapidly compute actuation policies with limited computational resources.

## Conclusion

The goal of this article is to realize rolling locomotion of compliant spherical tensegrity robots through their structural deformation. For this, we first introduced the concept of a step as a segment of rolling locomotion and classified it into different categories based on the geometry of the spherical tensegrity robots considered in this work. We also provided in detail the method to predict the deformations of spherical tensegrity robots given actuation commands based on the dynamic relaxation technique with kinetic damping.

Clearly, not all deformations would allow the tensegrity robots to make a step from one base polygon to another, and hence, some deformations are preferred to others for making a step. We defined actuation policies as the set of actuation commands that would result in these favorable deformations that can realize steps and precision rolling of tensegrity robots.

Two methods have shown to be successful in developing actuation policies enabling different types of steps. Both methods utilize the same heuristic that is defined based on a physical observation that the robot performs a step when it becomes unstable by placing its ground projection of the center of mass (GCoM) outside of its base polygon. To be precise, the heuristic is defined as the distance between GCoM and an edge of a base polygon serving as a rotation axis of a step. Since this physical condition for making a step is common to general spherical tensegrity robots, the developed methods are applicable to the robots beyond the ones introduced in this work.

The first method uses a greedy search algorithm, which we implemented in MATLAB to find actuation policies for different types of steps of the six- and twelve-rod tensegrity robots. This approach quickly finds actuation policies, and thus, it is suitable for checking the feasibility of steps or obtaining actuation policies that can serve as an initial solution to a more sophisticated optimization-based approach. Furthermore, this method is less computationally intense than MGMC, and it can be implemented on a resource-constrained on-board computer to (re-)compute actuation policies of the robots when the previous actuation policies become not valid anymore. We tested the obtained actuation policies on our hardware six-rod tensegrity robot and showed that the robot was successful in making desired steps from all base triangles in all directions.

The second method is based on a MGMC sampling. The method aims to find actuation policies that would improve the robustness of steps, and it achieves this goal by sampling a set of actuation commands and evaluating the resultant deformations over multiple generations to find suboptimal actuation policies and deformations that minimize the heuristic value. To demonstrate the method, we computed actuation policies for a CO-step of the six-rod robot and PD- and DP-steps of the twelve-rod robot with this approach. We then implemented the CO-step actuation policy on the physical robot and verified that the policy is indeed valid.

The comparison of the actuation policies by the greedy search and MGMC reveals that the latter would result in more reliable steps with less actuation energy. However, the greedy search policies are computed much faster with less computational burden.

Finally, we note that our methods are also applicable to a general class of spherical tensegrity robots beyond the robots introduced in this work. They can also be useful as a design tool. For example, if the degree of deformation is specified from a certain objective, then the number of actuators or the amount of actuation required can be computed using the presented methods. Moreover, the methods can be used to estimate the maximum ground inclination the robots can manage or to compute actuation policies to overcome a certain inclination, which is an ongoing research topic in the field.^58^

## Footnotes

^4^ Here, the term *node* is used in the context of a search algorithm. Each node can be thought as an abstraction of an actuation policy. Note that the term was also defined as a rod end in the previous sections, but its usage should be clear from the context.
